# Elemental Characterization of Romanian Crop Medicinal Plants by Neutron Activation Analysis

**DOI:** 10.1155/2017/9748413

**Published:** 2017-05-23

**Authors:** Daniela Haidu, Dénes Párkányi, Radu Ioan Moldovan, Cecilia Savii, Iulia Pinzaru, Cristina Dehelean, Ludovic Kurunczi

**Affiliations:** ^1^Institute of Chemistry Timişoara of the Romanian Academy, 24 Mihai Viteazul Bvd., 300223 Timișoara, Romania; ^2^Centre for Energy Research, Hungarian Academy of Sciences, 29–33 Konkoly Thege Miklós út, Budapest 1121, Hungary; ^3^Bio Vital Fares Laboratories, 50 Plantelor Str., 335700 Orăștie, Romania; ^4^Pharmacy II Department, Faculty of Pharmacy, “Victor Babeş” University of Medicine and Pharmacy, 2 Eftimie Murgu Sq., 300041 Timișoara, Romania; ^5^Pharmacy I Department, Faculty of Pharmacy, “Victor Babeș” University of Medicine and Pharmacy, 2 Eftimie Murgu Sq., 300041 Timișoara, Romania

## Abstract

The metallic elements concentrations of medicinal plants (coriander, dill,* Echinacea*, lavender, chamomile, mint, and plantain, used for phytopharmaceutical products), cultivated in unpolluted region, were analyzed by neutron activation analysis. The essential nutrients, macro-, micro-, and trace elements (K, Ca, Mg, Na, Fe, Mn, Rb, Sr, and Zn), potentially toxic elements (Al, As, Ba, Co, Sb, Cr, and V), and rare earth elements were monitored and were compared with those presented in the literature. An estimation of their contributions to intake and toxicity for a person was made, which revealed that (a) teas prepared from the examined plants represent useful contribution to the food provided intake of three essential macronutrients (K, Ca, and Mg); (b) the Cu, Mn, Rb, Sr, Zn, and rare earths levels are normal or low; (c) the quantities of As, Ba, Co, Sb, Cr, and V do not represent toxicological concerns; (d) the examination of the estimated Al and Fe quantities recovered in infusions in the conditions of usual daily tea consumption is below the Tolerable Daily Intake values. The strategy of cultivation of medicinal plants in unpolluted areas is efficient and beneficial. However, individual plants ability to concentrate preferentially certain elements suggests controlling the contamination level of raw materials.

## 1. Introduction

The preventive treatment mode and the identification of curative remedies for known diseases are not only a challenge but an imperative way to prevent and treat the evolution of possible diseases. In the development of medical advances, for a while, the discovered drugs (mostly coming from chemical synthesis) with their immediate action and desired therapeutic effects seized conventional medicine, reducing or even suppressing the influence of alternative medicine [1]. But once the chemically synthesized drug was understood in all its aspects, with therapeutic action, side and adverse effects, drug resistance, and the interactions between the active molecule and the dynamics of the human body have proved difficult to control easily. So nowadays the current trend is a return to nature, to known remedies, and among that to medicinal plants [1, 2]. The quality of these plants, in fact the raw materials for production of teas, syrups, tinctures, capsules, or other pharmaceutic formulas, is decisively influenced by the environment (soil, nutrients, pollution, etc.) where they are growing. All these forms of pharmaceuticals based on plants are included in the category of nutritional supplements, which are not a subject to strict control. Although medicinal plants are considered harmless by ordinary people, a closer look at the risk of accumulation and control of toxic metals or undesirable elements, evaluating content items is justified and desirable to be a criterion for their use [3, 4]. If the medicinal plants are harvested from the spontaneous flora, they may be contaminated by heavy metals [5, 6] because of where they were gathered. In this context, the contamination with known or unknown pollutants raises the hypothesis of crop cultivation of the medicinal plants, in places protected from pollution, an important extra argument besides the economic issues.

Concerning the quality and safety of the herbal products, the elemental composition of the raw plant material should be a required issue. Generally, the essential nutrients (K, Ca, Mg, Na, Fe, K, Mn, P, and Zn), micro- and trace elements (Co, Cr, Cu, Ni, Se, and V), and undesirable, potentially toxic elements (Al, As, Ba, Cd, Co, Cr, Hg, Ni, Pb, Sb, and Sn) are monitored in this context [7]. This endeavor is performed using mainly the following analytical methods: flame atomic absorption spectrometry, electrothermal atomic absorption spectrometry, inductively coupled plasma optical emission spectrometry, and inductively coupled plasma mass spectrometry. These methods involve digestion of the samples, with certain losses, an uncertainty in methods applied to release of the elements from their natural matrix, and also instrument and operator errors [7]. There are also methods that use only finely grinded and homogenized samples, without further preparation, and are nondestructive and with high sensitivity: prompt-gamma neutron activation analysis and neutron activation analysis (NAA). NAA is a specific and accurate analysis technique, recently classified as a primary ratio method by metrology [8, 9]. NAA is sensitive for trace elements (Cu, Zn, As, Se, Rb, Sr, Sc, V, Cr, Co, Ni, Zr, As, Sb, Ce, Ba, Hf, Ta, and W), for macroelements (Mg, Ca, Na, and K), for other elements (as Ti, Al, Fe, and Mn), and also for rare earth elements and is particularly suitable for medicinal plants. The analysis of medicinal plants and tea by this method has a long history [10–13] but it is not very abundant in trials because of the high costs and low affordability. However, the results obtained by this method are valuable and rewarding [14–17]. Already in 1996, the World Health Organization (WHO) has recommended NAA as analytical method applicable for plant materials [18]. Furthermore, it was also used in a proficiency test for heavy metal content (undesirable substances) in vegetable feed to establish the reference values [19].

The aim of the present study is the NAA of the elements contained by seven herbs cultivated in medicinal crops, away from any source of pollution, originating from the central part of Romania. Furthermore, these herbs are representative medicinal plants, are accessible and widely used through different pharmaceuticals forms or voluntarily by the entire Romanian population, and are exported also in Europe. The results will be compared with those presented in the specialized literature and will be analyzed from point of view of their contributions to intake and toxicity of the metals for humans.

## 2. Materials and Methods

### 2.1. Sampling and Sample Preparation

The plant material has been selected as representative specific medicinal plant crop that is currently in production in an uncontaminated area, near to Orăștie, Romania: peppermint, plantain, chamomile, lavender,* Echinacea*, dill, and coriander. The seven species of plants were obtained from cultures of 2015. Species and plant parts used for analysis were leaves:* Menthae folium* from* Mentha piperita L*., and* Plantaginis folium* from* Plantago lanceolata L*.; flowers:* Matricariae flos* from* Matricaria chamomilla L*., and* Lavandulae flos* from* Lavandula angustifolia Mill.*; the aerial parts:* Echinacea herba* from* Echinacea angustifolia DC.*; fruits:* Anethi fructus* from* Anethum graveolens L*., and* Coriandri fructus* from* Coriandrum sativum L.* As standard reference material, the WEPAL (Wageningen Evaluating Programs for Analytical Laboratories) IPE sample 205 of Tobacco (leaf-mixture)/*Nicotiana *Solanaceae from Netherlands [20] was used.

The raw material, air-dried plants (about 100 g each), as they are used in production of a range of products, were finely crushed with a manual grinder with ceramic knives and with ceramic mortar and pestle. No metal tools were used to avoid the risk of contamination. The samples were strained through a plastic sieve with a meshed bottom (0.5 mm diameter). The average sample was obtained by quartering [23]. Given the assay sensitivity, the process of crushing and homogenization of samples has great importance for accurate and precise results. Samples were subjected to drying in an oven at 105°C to constant weight. The weight loss (water and some volatiles) was 9.11%, 7.16%, 7.27%, 7.93%, 9.31%, 7.29%, and 7.28% for mint, plantain, chamomile, lavender,* Echinacea*, dill, and coriander, respectively. The samples obtained in this manner were delivered to the NAA. At the NAA laboratory a refining of the “drying” was carried on using independent samples (105°C, 4–6 hours, dry until constant mass). The weight loss (% volatiles) ranged between 0.24% and 0.79% for the analyzed plants and was 4.67% for the Tobacco leaf-mixture. Thus, the weights of the NAA samples were corrected with a mass factor of 1/[1 + (% volatiles/100)]. This value is considered as reference for the determined element contents, expressed as mg element/kg dry plant.

### 2.2. NAA

The neutron activation analysis was performed using the 10 MW Budapest Research Reactor [9]. Short-term and long-term types of irradiation were undertaken targeting radioactive daughter nuclei, with short or long half-lives, as described [24].

Thus, for the short-term irradiation, samples of 3.1 ÷ 8.5 mg were weighted, packed in Whatman 41 filter paper, pressed in pellet, and placed in cleaned polyethylene capsules and irradiated for 2 minutes. After irradiation, the sample pellets are repacked into inactive polyethylene and measured. A blank correction was performed for the Whatman 41 paper: the blank and the sample filter Whatman weights were always determined before sample preparation, and a subtraction was made for the “mass-factored” Whatman paper.

The 6-hour long-term irradiation was performed on samples of 44.3 ÷ 92.3 mg sealed in high-purity quartz ampules, 6 cm long, 6 mm diameter, Suprasil© AN, Heraeus. The Suprasil type quartz contains metallic trace impurities in concentrations ≤0.010 mg/kg (only for Ca ≤ 0.015), as stated in [25]. To remove surface contamination with some of the measured elements, the inner and outer surfaces of the ampules were etched in a mixture of hydrogen fluoride, acetic and nitric acid, and H_2_O_2_, washed with double distilled water, and dried (as described in [9]). The thermal equivalent flux parameters were 5.68 · 10^13^ and 2.00 · 10^13^ cm^−2^·s^−1^; alpha (the deviation of the epithermal neutron flux distribution) 0.003 and 0.002; *f* (the ratio of the thermal to epithermal neutron flux) 37.9 and 51.2; 4.48 · 10^12^ and 1.16 · 10^12^ fast neutron flux/cm^2^·s for the short-term and long-term activation, respectively. In short-term irradiation the Al, Ca, Cl, Cu, Mg, Mn, S, and V contents were evaluated, whereas As, Au, Ba, Br, Ce, Co, Cr, Cs, Eu, Fe, Ga, K, Na, Rb, Sb, Sc, Sm, Sr, Tb, Th, Yb, and Zn concentrations were investigated in long-term irradiation. The gamma-rays emitted from the samples were counted at 10 to 25 cm distances from an n-type coaxial HPGe with 36% relative efficiency [9] with 10 minutes of detection time, at 2-3 minutes after the short-term irradiation, and in two stages (a) with 20 minutes of detection time at 3-4 days after the long-term irradiation and (b) with 6 hours of detection time at 25–30 days after the long-term irradiation. Spectra evaluation was performed (i.e., peak fitting) with the Hyperlab 2013 software [26], described in [27]. Concentration calculation was performed using the *k*_0_-standardization method and the Kayzero for Windows software [28]. The given uncertainties are equal to 2 standard deviations including 3.5% systematical uncertainty (resulting mainly from the uncertainties in the measurement of terms from the definition of *k*_0_, [29]) plus spectra evaluation uncertainty, that is, area determination uncertainty.

## 3. Results

To verify the performance statistics of the applied NAA method, a certified reference material was used: IPE sample 205 Tobacco (leaf-mixture), from the WEPAL reference materials. The chosen performance statistics parameter is the *E*_*n*_ number in compliance with ISO 13528, 2015. The results are represented in Table 1. The means and the corresponding standard deviations for the reference material are extracted from the consensus table values (except for Al, for which the consensus value is “so-called total” and consequently the indicative value is used) presented in the Certificate of Analysis-IPE sample 205, WEPAL [20].

Although [30] in the section “The Ashing of Organic samples” considers arsenic loss by volatilization important only in presence of HCl and thus [31] does not include arsenic in the list of oven-dried tested trace elements regarding possible losses, we discuss this problem. After a careful survey of the relevant literature, the findings are as follows: the common terrestrial plants uptake mainly water soluble (polar or ionic) species of arsenic, even if they are methylated and considered as “organic” [32]. In the plant, As(V) can be reduced to As(III), and a great part of these species are complexed with glutathione, phytochelatins, and proteins [33, 34]. The dissociation of these complexes produces also ionic or polar arsenic species [35]. Speciation studies (e.g., [36] refers also to one of our plants, plantain) demonstrate that even the noncomplexed arsenic species are very polar and nonvolatile or ionic compounds (e.g., dimethyl arsenic acid, monomethylarsonic acid, trimethyl arsine oxide, tetramethyl arsonium ion, and arsenobetaine). Thus, a significant arsenic volatility and loss in the conditions of our sample preparation procedure are improbable. This is demonstrated also by the As *E*_*n*_ value in our Table 1.

Another potential problem might be the interference of an activated phosphorus isotope with that of aluminum, thus introducing errors (overestimation) in Al content determination. Following the rationale of Alfassi and Rietz [37], the contribution of the irradiated ^31^P to the ^28^Al signal can be determined. For a light water reactor (as the Budapest Research Reactor), using the mean of five values delivered by this reference 32.8 mg of P in the sample gives the same ^28^Al activity as 0.01 mg of Al. Picking up the corresponding phosphorus content of our plants from [38–46] can calculate the mean P concentrations (mg/kg), as follows: coriander 4041, dill 1745,* Echinacea* 2810, lavender 2810, chamomile 3635, mint 4046, plantain 4790, and Tobacco 4630 (from IPE 205) [20]. From these data, the following correction rates can be calculated for each of the plants: 4.07%, 0.34%, 1.80%, 0.01%, 0.33%, 0.13%, 0.06%, and 0.25%, respectively. The values are in accordance with those obtained by [47] for plant standards. The numbers are less than the relative standard deviations for Al from Table 2. Thus, the perturbation caused by ^31^P can be neglected. As a matter of fact, the *E*_*n*_ value for Al from Table 1 passed on the edge the ISO 13528, 2015 [22] test (see the Discussion).

The NAA results for the seven analyzed plants are presented in Table 2.

Although not all the elements present in Table 2 are monitored in Table 1, with a correctly implemented Kayzero assisted NAA is possible the reliable determination of the concentrations also for these elements [48]. As can be seen from [9, 49] at the Budapest Research Reactor the *k*_0_ procedure was successfully implemented and validated. The statistical validation from Table 1 also assures that the results for these elements in Table 2 must present good accuracy levels. An older and a newer reference exemplifies similar situations from the literature [50, 51].

## 4. Discussion

The absolute values of the normalized error *E*_*n*_ from Table 1 are smaller than 1, except for three elements for which they are very close to this value. With the critical value of *E*_*n*_ being 1 (ISO 13528, [22]), the NAA values passed or very nearly passed the test of performance statistics. Thus, the method can be used for determination of the element concentrations in the investigated plant samples.

**Figure 1:**
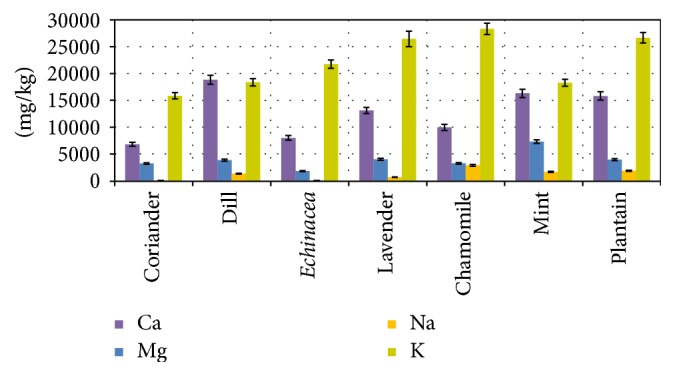
Selected macroelement (K, Ca, Mg, and Na) content in the plant samples.

Among the essential metallic macroelements [52], potassium is present in the largest quantity in the analyzed samples. For this element, comparable results were obtained for chamomile, mint, and coriander (air-dried plant material collected from “local bazaar”) by [43] using ICP-OES (Inductively Coupled Plasma Optical Emission Spectroscopy).  Figure 1 presents the concentration K for the seven plant samples determined by NAA, together with other three macronutrients: Ca, Mg, and Na.

Inspecting Figure 1, one can notice that for calcium and magnesium there is a visual similarity of the content ratio of these two elements in the plant samples. A similar behavior is reproduced for chamomile and mint in [43] and for* Echinacea* and mint in [39]. On the other hand, the K/Na ratio was used to characterize the diuretic activity of several medicinal plants. The value obtained for chamomile (615 : 1) by [53] is very different from that obtained using the data from Table 2 (≈10 : 1), but [43] confirms this last ratio.

Hereinafter the quantity of element retrieved in teas will be calculated using the percent transferred into the infusions/decoctions specified in the literature concerning the analyzed plant, taking into account the quantity of powdered tea plant used, if it is dried or not, and the concentrations from Table 2.

The amounts of K, Ca, and Mg transferred into teas from plant samples reported by [46] were between 51.6 ÷ 59.2%, 15.8 ÷ 35.7%, and 18.1 ÷ 26.5% for coriander, mint, and chamomile, respectively. Using these values and taking into account for the three plants the concentrations from Table 2, 50 ml tea prepared from 2.5 g of powdered plant (as was prepared in [46]) will contain between 20.8 ÷ 38.8 mg of K, 3.5 ÷ 13.2 mg of Ca, and 1.3 ÷ 3.3 mg of Mg intake. These amounts are far below the daily normative requirements and the recommendations provided by [52] for the three essential macronutrients but can represent a useful contribution to the food provided intake.

**Figure 2:**
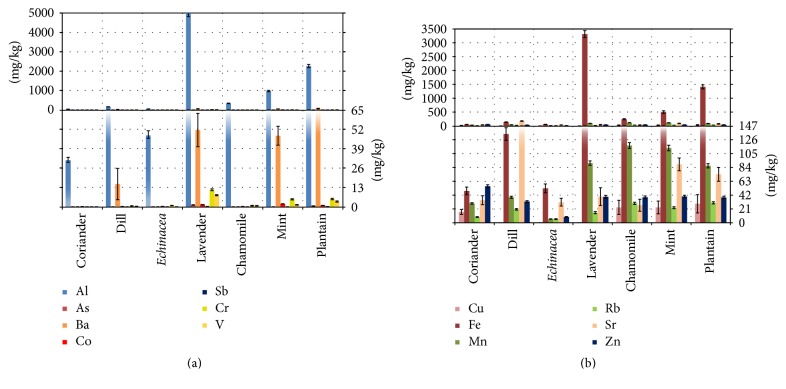
Diagrams of trace and ultra-trace (micronutrient) element concentrations for the seven plant samples; each element is represented in two concentration ranges: a higher one (left vertical axis and upper part of the chart) and a lower one (right vertical axis and lower part of the chart; the gradient fill suggests that the values are higher than the upper limit of this range): (a) elements with toxic or potentially toxic effects and (b) other elements.

Figure 2 presents diagrams of selected trace and ultra-trace [52] element concentrations for the analyzed medicinal plants. The first diagram (Figure 2(a)) contains toxic or potentially toxic elements and the second (Figure 2(b)) contains other elements. For comparison reasons and better clarity, the same elements are represented in two scales, a higher one and a lower one (see the figure's caption).

Being that any element may be toxic function of the intake quantity [52], the arsenic content of the analyzed plants is very low or under the limit of detection, as opposed to the results of [43, 54], on samples whose origin (spontaneous flora or cultivated) cannot be very clearly established. Furthermore, as stated by [54], for example, chamomile retains 32 ÷ 35% (mint only 12 ÷ 16%) of the arsenic content of the infused plant. The WHO provisional Tolerable Daily Intake (TDI) value of 0.0021 mg/kg body weight/day was withdrawn [55] and no new level was established. In the USA [56] the TDI is 0.0003 mg/kg body weight/day (0.021 mg/day for an adult of 70 kg). The highest arsenic concentration in Table 2 is retrieved for lavender, and with an infusion transfer of 35% a cup of tea using 2.5 g plant material contains 0.001 mg As, far below the accepted daily level for a person.

“Toxic elements” chromium, vanadium, and cobalt are clearly present, but not in high amounts, in the lower concentration range of Figure 2(a).

The chromium level in the analyzed plants (under 1 mg/kg, except mint, plantain, and lavender) in other papers is either approximately the same for lavender [42, 57] and for mint [43, 46] or greater for coriander, chamomile, and dill [42, 43, 45, 58]. These papers state Cr recoveries, depending on the preparation method used, in the infusions of 3.6 to 10.9% (coriander, mint, and chamomile, 2.5 g plant in 50 ml water, [46]) or 59 to 80% (chamomile, 2 g dry plant in 100 ml boiling or hot water, 5 minutes, [58]). From the data of [59], the calculated chromium transfer in boiling water (1 g of plant powder in 200 ml water) for mint is 23.5% and for chamomile 17.6%. Paper [57] claims that the lavender chromium is very aqueous soluble in hot water (during the distillation for lavender oil preparation). Even using the worst case of infusion release (80%), from 2.5 g of raw lavender sample from Table 2 (no percentage data for the infusion), 0.021 mg Cr is transferred in a cup of tea; the quantity is much smaller than the TDI of 0.245 mg, and the daily intake from mixed diet is only 0.061 ÷ 0.084 mg, all the quantities being calculated for an adult [52].

Other sources indicate the same vanadium concentrations as in Table 2 for mint and chamomile [43], somewhat smaller for lavender and greater for coriander and dill [42]. For vanadium in the USA, the Tolerable Upper Intake Level (equivalent with the TDI) is 1.8 mg/day for an adult [60]; [52] indicates 7 mg/day (0.1 mg/kg body weight). Paper [61] stated that a tea produced from one bag (1.85 g) of peppermint leaves (50 ml water at 95°C, for 15 minutes) contains 22.5% of the vanadium from the raw material. The infusion V release compiled by [7] is 1.4 ÷ 40.5%. Using the last value and the lavender V concentration from Table 2 a cup of tea prepared from 2.5 g raw plant powder contains 0.007 mg of vanadium.

Only mint samples revealed lower concentrations of cobalt than those from Table 2; coriander and chamomile contain higher Co levels (data from [43, 46]). The infusion transferred Co is 26.7, 68.3, and 15.3% for coriander, mint, and chamomile, respectively [46]. A mint tea prepared from leaves containing 1.82 mg/kg Co (Table 2) in the conditions mentioned by [46] will contain 0.003 mg cobalt. There is no official WHO established TDI value for cobalt intake [62], but 0.0014 mg/kg body weight (≈0.1 mg/day) was proposed by [63].

As a consequence of the last three paragraphs the chromium, vanadium, and cobalt concentration values from Table 2 are not alarming.

The metals with the most significant concentrations in Figure 2(a) are barium and aluminum.

Comparatively with the values from Table 2, the barium concentration levels reported in other papers are slightly higher (coriander, [43]), higher (coriander, [42]; chamomile, [64]), much higher (mint and chamomile, [65]) of the same order of magnitude (mint, [43, 45, 61]; lavender, [42]), or smaller (in plantain flowers, [66]). Only 18.2 ÷ 18.5% of Ba is extractable by infusion from the mint leaves (1 tea bag, 100 ml boiling water, 5 minutes, [45]) and 20% from chamomile flowers (0.5 g tea, 25 ml water, 30 minutes, [64]). The richest Ba content from the plants in Figure 2(a) presents plantain. Taking into account the maximum percentage value from the range mentioned by Pohl et al. (2016) [7] for Ba transfer, the extraction of a bag of 2.5 plantain ends with 0.099 mg Ba in the infusion. The assessed TDI of barium is 0.2 mg/kg body weight, that is, 14 mg daily intake for an adult [67]. Consequently, even in the extreme conditions used above, the concentrations from Table 2 are acceptable.

Seemingly the Al content from Figure 2(a) is of some concern, but different medicinal plants contain much more aluminum: the* Camellia sinensis* tea leaves over 30,000 mg/kg [68], some Moroccan plants 12265 ÷ 79152 mg/kg [69], green teas up to 13000 mg/kg, and black teas up to 27000 mg/kg [70]. For herbal teas, the aluminum content is generally lower than for black and green teas [71]. In the literature for the plants from Table 2, similar Al concentration values were recorded by [43, 46] (mint) and by [43, 64] (chamomile). At the same time, lower values were measured for mint by [44, 45, 59], for chamomile by [44, 46, 59], and for lavender and for dill by [42]. But the aluminum concentration is almost twice of that from Table 2 for plantain flowers in [66] and 14 times and 46 times greater for coriander in [42] and [46], respectively. The aluminum content in infusions depends on the duration of the extraction process: for eight different* Camellia sinensis* tea samples produced in four countries, the recovery was between 19.5 ÷ 49.7% for 2 minutes, 29.4 ÷ 60.2% for 5 minutes, and 33.2 ÷ 63.3% for 10 minutes [72]. Szymczycha-Madeja et al. [70] in a survey article register a range of 2.6 ÷ 42.5% of Al recovery for green teas and 2.6 ÷ 63.3% for black teas. For herbal teas, [71] indicates Al recovery values within 1.37 ÷ 3.61%. Likewise, for the herbal plants from Table 2, the aluminum extracted through infusion or decoction must cover a narrower range (the preparation conditions were already described for almost all references mentioned hereinafter). Indeed, for chamomile the values are 0.43% [64], 4.18% [46], and 15.1% [59]; for mint 0.16% [46], 1.66 ÷ 2.1% [70], and 17.6% [59]; for coriander 0.19% [46]. The plant rising apparently concerns inspecting Figure 2(a) is lavender. A cup of tea prepared from 2.5 g of lavender implying the value of 63.3% recovery (see above) is about 7.2 mg Al or using the maximum recovery value obtained among the herbal plants, about 2 mg Al. The first condition imposed here is severe (the highest percentage from the literature, 10 minutes of extraction time, very high leachability of Al from the tea matrix, Tetley tea, already in the first 2 minutes 44.9%); thus the real values are certainly smaller than 7.2 mg. In the chapter of Anke [52], the TDI for Al is 1 mg/kg body weight, 70 mg for an adult/day, or 20 mg/day if the assessed provisional tolerable weekly intake of 2 mg/kg body weight from [73] is used. The daily Al intake of adults with mixed diet reported by [52] is 5.4 mg for women and 5.8 mg for men. So, as stated in [74], some teas should be consumed with caution (e.g., letting tea steep no longer than 3 minutes), to not exceed the TDI threshold level. It can be mentioned also that an in vitro method suggests that only 4.8% of the Al from a tea infusion is potentially available for absorption [75], and rat in vivo experiments establish that only 0.37% is orally bioavailable [76]. Also, [77] suggests based on ingestion in healthy volunteers that “Al ingested from short-term tea drinking does not contribute significantly to the total body burden of this metal.” The facts described here suggest on the one hand that the Al concentrations from Table 2 are not unusual and on the other hand that the quantities recovered in infusions in the conditions of usual daily tea consumption are below the TDI value.

Besides the papers mentioned at the analysis of Figure 2(a), for comparison reasons concerning the metals represented in Figure 2(b), the following references were used: [38, 78–81].

Generally, the iron content of the plants from Table 2 in other references is higher for* Echinacea* and dill, smaller for lavender, approximately the same or smaller for mint and plantain, and the same, smaller, or higher for coriander and chamomile. In the case of lavender, the highest value from the references is 2.7 times smaller than that from Table 2. The Fe recovery by infusion for mint is 0.26 ÷ 6.84%, for chamomile 3 ÷ 14.61%, for* Echinacea* 6.43%, and for coriander 0.41%. The values are small or moderate; consequently, the concentration levels obtained for these plants and presented in Table 2 are not troublesome, especially as [70] classified Fe among the poorly extractable elements (below 20%). If the highest percentage of release mentioned above is used to estimate the Fe content of a cup of tea prepared from 2.5 g lavender, the result, 1.1 mg, is well below the TDI for an adult, 0.7 mg/kg body weight, that is, 49 mg for an adult [52].

In Table 2, the highest concentrations for Cu and Mn are retrieved for plantain and for chamomile, respectively. Other sources indicate for plantain slightly smaller Cu concentration levels and for chamomile similar, smaller, or higher Mn concentrations. The estimated Cu retrieval in infusions for mint, chamomile, and coriander ranges between 21.3 and 86.63%. This led to a maximum 0.038 mg of copper (chamomile, 69.58% retrieval, 2.5 g tea flowers, [46]) in a cup of tea, the TDI value being 0.175 mg/kg body weight, or 12.25 mg for an adult [52]. The literature mentions maximum percentage for manganese retrieved in infusion being 97.5% for chamomile and 40.2% for mint, meaning that using 2.5 g of the corresponding plant samples from Table 2 a cup of tea will contain about 0.28 mg and 0.11 mg Mn, respectively. The TDI value for an adult is 4.2 mg/day, that is, 0.06 mg/kg body weight [82].

The normative requirement of zinc for women and men is up to 6 ÷ 8 mg/day, and the TDI is 0.600 mg/day body weight [52]. Similar Zn concentrations with those from Table 2 are retrieved in the literature for lavender and mint. Coriander exhibits smaller Zn levels, and higher or smaller values are mentioned for* Echinacea*, dill, and chamomile. The infusion concentrations of Zn represent 21 to 73% of the metal content of the plant powder used. This means implying the concentration values from Table 2 (≈40 mg/kg), up to 0.07 mg Zn per cup of tea.

The Sr concentrations from Table 2 are the same or smaller in the monitored papers, but the element is mentioned between the poorly extractable ones (<20%) by [44]. Considering 20% retrieval for dill (the highest Sr content in Table 2), one can consider 0.08 mg intake for 2.5 g of plant infusion, which is much below the TDI of 9.1 mg/day for an adult [83].

Although rubidium seems to be most important for human health [52], very few data are mentioned in the literature for the plants from Table 2. The concentrations for chamomile are similar and for mint smaller. Concerning the total metal in the tea samples (Lipton Yellow Label, 50 tea bags) 85% is soluble by infusion [84]. Using the Rb content for plantain (Table 2) and the 85% retrieval, 2.5 g of plant, a cup of tea will contain about 0.06 mg of rubidium. The Rb TDI is 2 mg/body weight [52].

Consequently, the metal concentrations presented in Figure 2(b) do not cause problems in what regards the safety for consumption of teas prepared from the corresponding plants.

Analyzing Figure 2 as a whole, a parallelism between the concentrations of Al and Fe is found. That is not surprising because when the metal concentrations of seven medicinal herbs (among these peppermint and chamomile), each one from two producers, were analyzed, a higher than 0.8 positive correlation coefficient was calculated between the concentrations of aluminum and iron [44]. Also, [85] discovers a positive correlation between Al and Fe (*r* = 0.74) for twelve commercial bagged black teas. The metal content for these elements in the plant samples from Table 2 (see also Figure 2) is in order lavender > plantain > mint > chamomile. This is in line with the literature, which has recorded several strategies of phytoremediation of contaminated soils using lavender crop [86], peppermint [87], plantain [88], and even chamomile [89]. It can be mentioned that the metal accumulation in lavender inflorescences does not contaminate the lavender oil produced from these [90].

**Figure 3:**
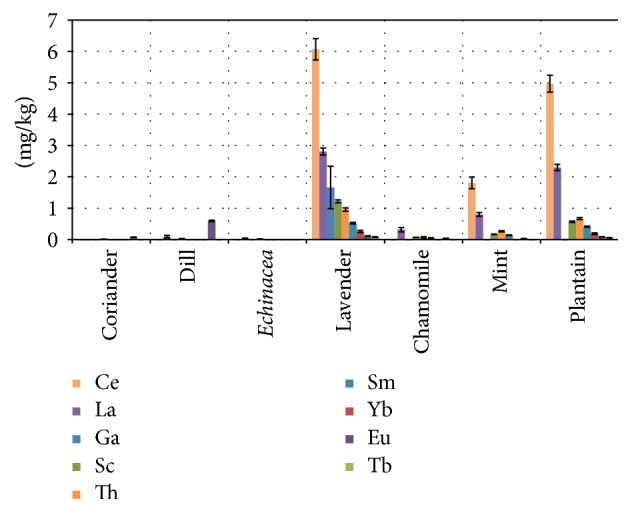
Concentrations of the rare earths in the plant samples.

Rare earth level determinations in medicinal plants are rather scarce in the literature. The concentrations of rare earths in the samples from Table 2 confirm the accumulation capacity of some of the plants and also the concentration order discussed above.  Figure 3 demonstrates clearly this fact.

The accumulation is more pronounced for the aerial part of the plants, than for the roots [91]. Paper [92] reports concentration values for cerium and lanthanum smaller for chamomile leaves than those from Table 2 and Figure 3 (here for flowers) and similar for* Mentha piperita* shoots (leaves in this paper), but much higher for* Mentha pulegium *leaves. In the case of* Plantago major*, a similar species with* Plantago lanceolate*, the reference indicates similar concentration for Ce and greater concentration for La. The same paper records for the infusion/decoction of the mentioned plants transfer factors lower than 10%. A cup of tea prepared from 2.5 g lavender (highest concentration in Table 2) will contain using a transfer of 10% 0.0014 mg Ce or 0.0006 mg La. The acceptable daily intake is 0.6 mg/kg body weight for rare earth oxides in [93] that means 0.5 mg/kg body weight for the metals (42 mg per person of 70 kg/day oxide or 35 mg metal). These circumstances suggest that the rare earths concentrations from Table 2 are normal and acceptable from toxicological point of view.

Lanthanides reveal a 0.01 ratio of the water dissolved element and the concentration in soil [94]. Soluble fractions of these elements from soil are absorbed by plants. The abundance of cerium, as average concentration in the soil, is 60 mg/kg and of lanthanum 30 mg/kg [95]. Lavender blossoms concentrate the largest quantities of rare earth elements. The concentration ratio between rare earth elements present in this plant (Table 2) and their concentration in soil is 0.1, equal for each of the analyzed elements (Ce, La, Sm, Eu, Tb, Th, and Yb). This constant ratio demonstrates a specific behavior, by the absence of the preference for some of the elements, and is 10 times higher than the solubility ratio mentioned above. This fact further evidences the bioaccumulation capacity of lavender.

## 5. Conclusions

A comparison with the element content of the plants originated from spontaneous flora and the plants analyzed here is difficult to perform, because only few papers declare clearly the nature of sample provenance. Only [59] states that the analyzed plants originate from the spontaneous flora. One can only surmise that at least part of the analyzed samples in [42, 43, 46, 66] were harvested from the spontaneous flora, because the plants were purchased from the “local bazar or market” or were “plants growing in Turkey.”

The comparative survey of these references and Table 2 led to the following conclusions: (a) the magnesium and calcium content (elements with normal daily intake below the requirement) are higher in the present study for chamomile and mint and the same for lavender; (b) the Fe and Zn level (marginally deficient elements in the daily food intake) are approximately the same, except lavender, for which the Fe content is higher in Table 2; (c) the toxic or potentially toxic elements present generally greater concentrations in the plants from spontaneous flora: As (chamomile), Al (coriander and plantain), Ba (chamomile, coriander, and dill), and Cr (chamomile and dill). Exception is the Al from lavender which is at higher level in Table 2, owing probably to the properties of the soil of the region where the plants were cultivated. Thus, the strategy to cultivate the medicinal plants choosing unpolluted area is practical and efficient.

The ability of individual plants to concentrate preferentially certain elements or group of elements suggests the need of controlling the level of some contaminants (e.g., aluminum) in herbal supplements. The same phenomenon suggests labeling of the products with higher content of potential contaminants, drawing the attention to limit the steeping of tea samples for short time.

## Figures and Tables

**Table 1:** IPE sample 205 Tobacco reference material [20] data (consensus values) and the NAA *E*_*n*_ performance statistics parameter^*∗*^.

Element (mg·kg^−1^)	Reference mean	Std. dev.	Measured mean	Uncertainty	*E*_*n*_
As	0.415	0.069	0.48	0.16	0.37
Ba	23.0	3.16	32.94	9.34	1.01
Br	99.0	9.39	102.8	3.7	0.38
Cl	4600	326	4207	155	1.09
Co	0.302	0.0471	0.34	0.03	0.68
Cr	6.4	0.964	7.51	0.43	1.05
Cs	0.0608	0.01314	0.05	0.01	0.65
Cu	12.3	1.18	17.18	7.36	0.65
Fe	330	37.7	366.5	19.7	0.86
K	54900	3600	56150	2050	0.30
Mg	4710	326	4930	194	0.58
Mn	296	21.8	302.7	10.9	0.27
Na	260	55.9	301.0	16.8	0.70
Al^*∗∗*^	388	180.3	566.5	19.8	0.98
Ca	30700	1970	32056	1249	0.58
Rb	26.5	2.92	28.28	1.71	0.53
Sb	0.222	0.0507	0.24	0.02	0.33
Sr	83.1	10.55	96.17	11.10	0.85
Zn	187	14.0	201.6	7.8	0.91
V	0.768	0.145	0.94	0.13	0.88

^*∗*^Std. dev.: standard deviation; measured mean: resulting from evaluation of the quantity of the element using several energy peaks from the gamma ray spectrum of the respective element (Kayzero for Windows, 2005) [21]; uncertainty: see Section 2.2; for the normalized error *E*_*n*_, see ISO 13528, 2015 [22]. ^*∗∗*^Indicative value.

**Table 2:** Concentration of the elements in representative medicinal plants as obtained by NAA: the values are means (mg·kg^−1^) and the corresponding relative standard deviations, RSD (%)^*∗*^.

Plant	Coriander		Dill		*Echinacea*		Lavender		Chamomile		Mint		Plantain	
Al	31.47	5.5	155.44	4.1	48.48	5.2	4996.71	3.6	334.88	3.9	975.55	3.7	2261.33	3.6
As	<0.10	—	<0.09	—	<0.08	—	1.16	10.9	<0.41	—	<0.12	—	0.42	39.6
Au	<0.00	—	<0.01	—	<0.00	—	<0.00	—	0.04	11.9	<0.01	—	<0.00	—
Ba	<6.9	—	15.18	69.5	<7.74	—	51.45	21.7	<8.16	—	47.65	13.3	75.12	11.9
Br	19.47	3.6	37.15	3.6	2.87	4.1	8.84	3.9	21.73	3.7	20.08	3.7	62.48	3.6
Ca	6787.58	5.5	18830.62	4.5	7990.88	5.3	13113.55	4.4	9944.35	5.7	16295.94	4.7	15807.76	4.9
Ce	<0.41	—	<0.31	—	<0.25	—	6.07	5.7	<0.43	—	1.81	10.3	4.97	5.4
Cl	553.02	4.2	5747.27	3.7	3897.67	4	996.93	4	8210.21	3.8	3731.17	3.8	4080.99	3.8
Co	0.04	31.7	0.16	6.9	0.13	11.6	1.3	4.4	0.1	13.6	1.82	4	0.8	4.3
Cr	<0.35	—	0.45	37.4	0.77	17.7	11.36	5.4	0.66	36	4.94	6.2	5.11	6.4
Cs	<0.02	—	0.03	30.6	<0.02	—	0.73	5.8	0.07	19.8	0.11	13.4	0.27	7.8
Cu	16.5	22.8	<24.81	—	<15.35	—	<31.62	—	23.36	45.5	23.3	41.6	28.9	48
Eu	0.08	5	0.59	3.7	<0.00	—	0.11	5.6	0.04	10.9	0.03	10.6	0.09	5.3
Fe	48.32	12.1	134.6	7.1	52.25	12.8	3315	3.8	239.1	8.1	498.2	9.3	1409	5.1
Ga	<0.36	—	<1.62	—	<0.36	—	1.66	40.9	<2.65	—	<1.36	—	<1.92	—
K	15840	3.7	18370	3.7	21740	3.6	26450	5.4	28330	3.7	18270	3.7	26670	3.7
La	<0.02	—	0.09	46.1	0.03	26.7	2.81	4	0.31	22.2	0.8	7.2	2.3	4.3
Mg	3203.3	4.1	3842.72	4.5	1785.74	4.8	4001.01	4.6	3243.33	4.8	7298.65	4.3	3941.71	4.7
Mn	29.41	3.9	38.85	3.9	5.66	5.5	90.53	3.7	117.25	3.7	113.43	3.7	86.56	3.8
Na	62.29	5.1	1330	5.6	26.39	4.7	653.1	6.2	2875	5.8	1649	6.1	1853	5.6
Rb	8.7	7.7	20.39	5.8	5.55	11.4	15.34	10.3	29.56	5.9	22.97	6	30.25	5.4
S	<4050	—	<6745	—	<4258	—	<7565	—	<6883	—	8866	39.5	9645	50.3
Sb	<0.03	—	<0.10	—	<0.02	—	0.15	18.9	<0.04	—	<0.04	—	0.06	41.3
Sc	0.01	11.3	0.03	4.9	0.01	9.2	1.22	3.7	0.07	4.5	0.17	4	0.57	3.7
Sm	<0.01	—	<0.01	—	<0.01	—	0.52	4.6	0.04	33.4	0.13	8.6	0.41	4.5
Sr	34.28	19.6	174.9	6	31.25	18.5	39.7	34	26.65	34.3	88.61	10.9	73.57	14.3
Tb	<0.01	—	<0.01	—	<0.01	—	0.08	15.2	<0.01	—	<0.01	—	0.05	12.6
Th	<0.03	—	<0.03	—	<0.02	—	0.96	5.4	0.07	29.6	0.26	8.1	0.67	5.1
V	<0.08	—	0.23	47.3	<0.07	—	7.73	5.1	0.61	28.6	1.24	14.4	3.47	8.3
Yb	<0.07	—	<0.05	—	<0.04	—	0.25	13.6	<0.07	—	<0.04	—	0.18	12.9
Zn	55.31	4.2	32.21	4.6	8.81	8.1	39.32	5.4	38.73	5	39.97	4.7	38.45	4.5

^*∗*^Mean: resulting from evaluation of the quantity of the element using several energy peaks from the gamma ray spectrum of the respective element (Kayzero [21]); RSD% calculated from uncertainty: see Section 2.2; < under the limit of detection (specified as a value).

## Abbreviations

ICP-OES: Inductively Coupled Plasma Optical Emission Spectroscopy
NAA: Neutron activation analysis
TDI: Tolerable Daily Intake
WEPAL: Wageningen Evaluating Programs for Analytical Laboratories.
